# Conducting Couple Interviews in Health Research: Methodological Lessons from Later-Life Caregiving Dyads

**DOI:** 10.3390/healthcare14070889

**Published:** 2026-03-31

**Authors:** Katharina Niedling

**Affiliations:** School of Public Health, Bielefeld University, Universitaetsstrasse 25, 33615 Bielefeld, Germany; katharina.niedling@uni-bielefeld.de

**Keywords:** qualitative research, couple interviews, dyadic interviewing, home care, informal caregiving, long-term care, spouses, couple, chronic illness, home-based care

## Abstract

**Background:** Qualitative health research increasingly emphasizes relational and interactional processes in illness and caregiving; however, joint interview formats remain methodologically under-theorized. This article advances a relational and power-sensitive reconceptualization of the couple interview by conceptualizing the interview encounter itself as an interactional site in which caregiving relations become observable in real time rather than merely reported retrospectively. **Methods:** The article draws on seven in-home couple interviews with long-married older heterosexual couples in Germany, in which one partner provided long-term home-based care for the other. The analysis applies the Documentary Method to reconstruct jointly produced meanings, collective orientations, and the micro-interactional dynamics of the interview situation itself. **Results:** The analysis shows that couple interviews provide a distinctive methodological lens for studying dyadic caregiving by rendering co-narration, negotiated speaker roles, “we”-positioning, speaking-for-the-other, and embodied coordination analytically visible. Interactional asymmetries, interruptions, and situational role shifts thus emerge not only as challenges but as epistemic resources for reconstructing caregiving relationships and power dynamics. Based on this analysis, the article develops a three-part practice-oriented methodological toolkit comprising relational interviewing strategies, moderation practices, and systematic observation and documentation markers. **Conclusions:** By reframing the couple interview as an interactional event and specifying analytic markers and conduct strategies, this article makes an explicit methodological contribution to dyadic qualitative health research, particularly in sensitive later-life caregiving contexts.

## 1. Introduction

Qualitative health research is increasingly focusing on the interactive negotiation processes within social relationships, as they have a decisive influence on how illnesses are experienced and everyday care is provided [1,2]. Accordingly, the analysis of interactions and communicative practices that are used to interpret and cope with illness and to organize care arrangements in everyday life is becoming increasingly important [1]. This applies, in particular, to chronic and care-intensive illness situations, in which coping with illness is not to be understood as an individual event but as a relational and interactive process that systematically involves relatives, especially partners [3]. At the same time, older couples are often characterized by a long shared relationship history in which stable interaction patterns have developed As a result, partners tend to play a particularly central role in illness- and care-related demands [4].

Despite the increasing interest in interaction dynamics and dyadic health processing, qualitative studies in the health sector continue to focus predominantly on individual interviews (see, for example, ref. [5]). This also applies to research fields that focus on older people and people in need of care. Collaborative interview formats such as the couple interview, on the other hand, are rarely used and have hardly been systematically methodologically analyzed to date. This means that an important potential remains unutilized. Particularly in long-term relationships between older adults or people in need of care, a couple interview enables access to jointly constructed interpretations, interactions, and negotiation processes for coping with and processing chronic illness. These aspects can only be captured to a limited extent through individual interviews.

In addition to the problem that couple interviews are rarely used, there is also a lack of guidance in the literature [6] on how to conduct them. Although individual studies have addressed the specific ethical and methodological challenges of joint interviews (see refs. [7,8,9]), systematic methodological reflections on how couple interviews can be planned and conducted remain rare. This article therefore examines the methodological opportunities and challenges that arise from interviewing both partners at the same time, with a particular focus on interview design and implementation. Drawing on an empirical study with older couples in which one partner requires care, this article uses this empirical material as a methodological case. It reflects on the challenges and opportunities of conducting couple interviews and develops practice-oriented methodological recommendations. By analyzing the interview situation itself as a site of data production, the article argues that methodological challenges in couple interviews—such as asymmetries, interruptions, and situational role shifts—can be understood as epistemic resources for qualitative analysis rather than mere obstacles.

### 1.1. The Couple Interview as a Dyadic Survey Method

The couple interview is an interview form with elements of autobiographical-narrative interviews and group discussion methods [10,11]. As a specific variant of the dyadic interview [12,13], the couple interview is conducted with partners in a romantic relationship and conceptualizes the couple relationship itself as the primary unit of analysis. This interview setting enables the observation of interactional dynamics that emerge through the simultaneous presence of both partners and the interviewer. Discussing the relationship and shared experiences in the presence of both partners allows interactional dynamics to become observable within the interview setting and to be analytically reconstructed.

In contrast to individual interviews, couple interviews aim to capture jointly produced meanings. The focus lies on the *in-between*, that is, on meanings that are co-constructed in interaction rather than individually articulated. As the couple interview is oriented toward jointly generated meanings, it is crucial to create conditions in which couples can communicate with one another in a relatively natural manner [14], p. 110. Particular attention should be paid to moments in which differences arise and to how these differences are negotiated within the interaction [14], p. 110. By interviewing both partners together, fragmentary insights into the dynamic inner workings of this social relationship can be gained. The couple interview thus provides deeper insights into the internal constitution of the couple and their everyday practices, as interactive negotiations unfold during the interview, for example, regarding thematic responsibilities and speaking rights [15]. In contrast to other dyadic settings, the couple interview addresses an intimate relationship [6]. Various interview-based methods, from structured guided interviews and problem-centered interviews [16] to narrative or (couple) biographical interview forms [17], are suitable for generating such shared narratives.

The couple interview is grounded in the interpretive paradigm of qualitative social research, conceptualizing the couple as an independent unit of analysis with its own social reality.

### 1.2. Conceptual and Theoretical Determination of the Couple Relationship

For the conceptual determination of couple relationships, the following section draws on Karl Lenz’s (2009) [18] definition, which characterizes couple relationships as social relationships in which a sense of mutual belonging, a shared identity (a “we-feeling”), and ritualized practices that stabilize the partnership are constitutive. According to Lenz [18], couple relationships are characterized by a collaborative relationship culture in which a shared reality is created and maintained through communication and negotiation. Over time, couples negotiate shared norms and establish a culture of intimacy, which also serves to demarcate the relationship from the outside world. Neither in groups, families, nor in arbitrary dyadic interview constellations can one readily assume the existence of a shared and durably stabilized interactional order (nomos) [6]. As Hirschauer emphasizes [19,20], social order is not presupposed but produced situationally in and through the enactment of interaction [19,20]. Unlike groups, which are often organized around specific topics or occasions and may persist independently of individual members, couple relationships are based on an exclusive dyadic bond with a shared relational history [6]. Over the course of repeated interactions, stable expectations, roles, and a specific nomos emerge.

According to the couple psychologist Jürg Willi (2012) [21], the success of a couple relationship is grounded in egalitarian roles within the partnership. From the perspective of Karl Lenz [18], particularly in long-term relationships, there is often a “high degree of commitment (exclusivity)” as well as an “increased level of attentiveness” within the relationship. The continuation of the couple relationship can therefore create stability and security for both partners.

### 1.3. Use of Couple Interviews in Health Research

In health research, interviews with caregiving and care-receiving dyads are increasingly used to examine processes of shared illness management. As these dyads often consist of intimate partners, the couple has emerged as a central unit of analysis. Particularly when studying older couples coping jointly with illness and care, couple interviews provide a practicable means of accessing sensitive experiences, as care recipients may be unwilling or unable to participate without their partner present. Often, these partners are themselves of advanced age and affected by functional limitations or illness, which constitutes an additional characteristic of this research setting.

There is a growing body of studies employing couple interviews in the field of health research, especially with regard to topics that affect or involve both partners, such as chronic illness and illness management [22,23]. Previous studies using couple interviews in health research demonstrate how partners co-construct illness experiences, negotiate roles and responsibilities, and coordinate illness management in interaction [24,25,26]. These studies highlight that the joint interview setting itself enables the observation of dyadic processes as they unfold, including the negotiation of dependency, autonomy, and changes in the couple relationship.

## 2. Materials and Methods

The present article draws on a qualitative study of caregiving relationships in later life. The substantive focus of that study was how long-married couples shape and experience home-based care situations. However, in this article, the empirical material serves as a basis for methodological reflection. In order to adequately capture these dyadic perspectives within a health-related context, the couple interview was employed as the primary method of data collection.

The following section outlines the methodological approach of the study, alongside key aspects of data collection. This includes the recruitment of couples, the study design, the interview constellation and procedure of the couple interviews, as well as a brief presentation of the results to contextualize the subsequent analysis.

### 2.1. Recruitment and Sample

Within the framework of the study presented here [4], couple interviews were conducted with heterosexual, long-married couples in Germany in which one partner provided care for the other. The aim was to explore what it means for a couple relationship when care dependency emerges within a long-term marriage and a permanent caregiving structure becomes established in everyday life. Participants were primarily recruited from couples or caregiving partners who had taken part in caregiving courses offered as part of the project “Familiale Pflege unter den Bedingungen der G-DRG” [Family Care under the Conditions of the G-DRG System] [27]. The couples were invited to participate either by me, the author of this article, or by the nursing professionals who facilitated the courses. Participants were informed both verbally and in writing about the aims, subject matter, and procedure of the study prior to participation. The ethical acceptability of interviews that had already been conducted was retrospectively confirmed by the Ethics Committee of Bielefeld University.

### 2.2. Study Design and Setting

Within the framework of the study, seven couple interviews were conducted with long-married older couples in which one partner provided care for the other. The interviews were conducted between December 2018 and June 2020 in the couples’ homes. Couple selection followed predefined inclusion criteria: both partners were aged 65 years or older, lived in a long-term marital relationship, and shared a household. In addition, a permanent home-based caregiving arrangement was required, in which one partner was identified as care-dependent and the other as the primary caregiver.

In two cases, the caregiving partner was male; in five cases, female. The duration of the marital relationships ranged from 30 to 63 years, while the duration of the caregiving relationships ranged from 1 to 30 years. The couples reported various age-related functional limitations and chronic conditions. Participants ranged in age from 65 to 95 years, and the couples had diverse educational backgrounds.

### 2.3. Development of the Interview Guide

The development of the interview guide followed several stages. In a preliminary study, three group discussions [28] were conducted with nursing professionals who supported family caregivers within the project “Family Care under the Conditions of the G-DRG System” and who possessed extensive contextual knowledge of the target group. During the group discussions, the nursing professionals were asked to share their assessments of the living situations, relationship constellations, and challenges faced by older couples in caregiving relationships. The themes identified were translated into an interview guide and subsequently refined through three pretests. The final guide included topics such as relationship history, role and task distribution, caregiving arrangements, and everyday organization, as well as changes in closeness, physicality, and intimacy. Methodologically, the couple interviews followed the logic of problem-centered interviews [16], combining thematic guidance with biographically oriented narrative stimuli in order to encourage extended, jointly produced accounts.

### 2.4. Data Analysis and Analytical Orientation

The interview data were analyzed using the Documentary Method [29,30]. This interpretative–reconstructive approach aims to reconstruct collective orientations and habitualized patterns of action. It is particularly well suited for the analysis of interactional processes and jointly produced meaning in couple interviews. To enhance methodological transparency, the article briefly addresses the potential risk of analytical circularity in reconstructing interactional asymmetries. To avoid presupposing asymmetry and subsequently “finding” it in the data, descriptive interactional observations are separated from interpretive claims. The analysis first focuses on sequentially observable conversational features (e.g., turn-taking, interruptions, speaking-for-the-other) without immediately framing them in terms of dominance or power. Only in a second step were these markers interpreted in relation to relational order, systematically considering alternative readings (e.g., support vs. gatekeeping) and countervailing instances. Regarding content, the interviews addressed, among other things, changes in the couple relationship in the context of age-related care dependency. These included aspects such as the increasing influence of illness on everyday life, renegotiations of roles and tasks, experiences of closeness and distance, and ongoing efforts to maintain the partnership. Against this background, the present study investigates older, long-married couples in which one partner provides care for the other, using couple interviews to explore how caregiving arrangements and relationship dynamics are jointly constructed in the context of long-term marital biographies. The substantive findings of the study regarding caregiving dynamics and relational transformations in later life are reported elsewhere [4]. In contrast, the present article does not aim to contribute additional empirical findings but to systematize methodological insights derived from conducting and analyzing the couple interviews.

## 3. Results

The following section presents the methodological findings derived from the empirical material. Section 3.1 summarizes the recurring interactional patterns across the couple interviews as the core empirical basis. Section 3.2 and Section 3.3 then show how these patterns shaped data collection in practice (interviewer positioning; interview structure and questioning; duration and setting; observation/documentation). While the broader study investigated caregiving dynamics and relational transformations in later life, the present article focuses exclusively on the methodological implications of conducting couple interviews in this context.

### 3.1. Core Empirical Methodological Findings

The analysis of the couple interviews revealed a series of recurring interactional patterns that constitute the core empirical foundation of this methodological contribution. These patterns do not primarily concern substantive statements about caregiving but instead highlight the interactional conditions under which such statements are produced. They relate to the ways in which relationship and care relations, and their situationally observable asymmetries, are interactionally produced, stabilized, or contested within the interview itself.

First, a consistent pattern of co-narration emerged. Memories, biographical events, and interpretations were rarely developed by one partner alone. Instead, they were jointly constructed through additions, completions, specifications, or corrections. These co-productive sequences made visible that shared past experiences and collective interpretations do not simply “exist” as given entities but are situationally negotiated in interaction.

Second, speaker roles and epistemic authority were continuously negotiated, for example in who initiated, corrected, specified, or claimed interpretive authority. Particularly in discussions of caregiving arrangements or relationship history, speaking rights were situationally recalibrated rather than statically distributed.

Third, the recurring pattern of “speaking-for-the-other” was observed. This practice functioned, on the one hand, as a supportive strategy, particularly in cases of cognitive limitation or difficulties in articulating thoughts. On the other hand it operated as a form of narrative gatekeeping through which alternative framings or interpretations were anticipated or constrained. In this way, the interviews made interactionally visible how care, protection, and potential forms of disempowerment may become intertwined.

Fourth, in passages marked by conflict or asymmetry, couples frequently engaged in explicit “we”-positioning. Particularly when caregiving-related dependency was thematized, partners often reaffirmed their couple identity (e.g., “We are a couple”). Such sequences demonstrate that equality and partnership are not simply presupposed but are actively defended or redefined under conditions of vulnerability.

Fifth, the home-based setting and situational interruptions (e.g., phone calls, answering the door, caregiving tasks) rendered forms of embodied coordination and situational role shifts directly observable. Care relationships were not merely described but were practically enacted within the interview. In these moments, it became evident how strongly caregiving structures everyday life and how attention, responsibility, and participation shift situationally.

Taken together, these patterns constitute the central methodological findings of the article. Given the small and socially specific sample, they are formulated as a proposition grounded in the present cases that future work with more diverse dyads may further examine.

### 3.2. Challenges of Data Collection Using Couple Interviews

Conducting the couple interviews with older partners in caregiving relationships revealed a number of methodological challenges that influenced both the interactional dynamics of the interviews and the resulting data. The following section reflects on these challenges as they emerged in the research process.

#### 3.2.1. Interviewer Positioning

I conducted the couple interviews alone, which entailed specific demands on the interview process. Beyond structuring the conversation and guiding the questions, I was required to continuously observe and moderate the interaction between both partners. Attending to non-verbal dyadic dynamics—such as role distributions, subtle forms of dominance, or expressions of mutual support—proved particularly challenging, as the analytical focus was not on a single interviewee but on the ongoing interaction between partners. Conducting the interview alone also concentrated interactional authority in one person. Through turn allocation, follow-up questions, gaze, and topic shifts, I inevitably co-shaped which perspective became hearable and which remained backgrounded.

In addition, structural differences between partners had to be taken into account. Interviewees often differed in their communicative resources and familiarity with interview situations, which shaped the course of the conversation. In some cases, one partner assumed a dominant role, while the other withdrew or remained in the background, for example when one partner began speaking on behalf of both: “Yes, if I may start—our relationship has of course changed…”. Such “speaking-for-the-other” functioned as one instantiation of the recurring pattern described in Section 3.1 (supportive assistance vs. narrative gatekeeping), with direct consequences for whose perspective became hearable in the moment.

Further challenges concerned the dynamics of proximity and distance between me, as the interviewer, and the interviewed couples. These dynamics were shaped by social characteristics such as age, biographical experiences, or similarities in everyday life. While shared characteristics fostered trust and openness, they simultaneously required reflexive distance [31]. Age differences between me and the couples influenced these dynamics. I was frequently addressed as “a young woman” or “at your age,” positioning me as biographically junior while simultaneously attributing to me institutional–epistemic authority as a researcher. This ambivalence could shape both deference and resistance in the interview encounter.

Within the health- and care-related context of this study, proximity and distance were also produced situationally. The couples’ shared experiences of illness generated a close internal mode of communication from which I remained excluded. This constellation created a particular vulnerability in the interview situation and limited immediate access to taken-for-granted interactional routines. In power terms, this meant that “access” to shared routines was negotiated in situ. Couples could include me through explanation and teaching, or maintain boundaries through shorthand references and mutual alignment.

#### 3.2.2. Interview Structure and Questioning Practices

As the couple interview constituted an interactional situation of joint meaning-making rather than the sum of two individual interviews, this posed challenges for question formulation. Questions therefore had to be phrased and addressed verbally and non-verbally so that both partners felt equally addressed and could freely decide who would initiate the narrative. To this end, an open-ended narrative-generating opening question was used that allowed different forms of negotiation regarding who would initiate the narrative: “Tell me about how you became a couple and how things have developed between you to date?” Subsequent narrative-generating questions were addressed to both partners jointly, as the dyadic production of narratives constituted the primary analytical focus. One such question was, “What rituals do you share with each other as a couple?” Nevertheless, selective individual addressing became necessary when one partner dominated the interaction for an extended period. However, these individual follow-up questions had to be embedded in such a way that ongoing shared narrative processes were not interrupted or disrupted. This posed a further methodological challenge. To address this, questions were briefly directed to one partner while remaining relationally framed and immediately reintegrating into the dyadic exchange. For example, the partner was asked: “Why do you care for your wife/your husband?” This formulation invited individual reflection while simultaneously orienting the response toward the relationship and the shared narrative.

For interviewees with cognitive impairments, questions were additionally formulated in easily understandable language [32] and supplemented with examples in order to ensure their participation in the conversation.

A further challenge consisted in addressing sensitive topics in a way that established a safe conversational setting and fostered trust in the interviewer’s careful and responsible handling of the couple’s accounts. This required particularly sensitive question wording as well as close attention to non-verbal signals within the couple’s interaction. Accordingly, participants were asked: “If you feel comfortable sharing, have there been phases in your relationship that you experienced as particularly challenging?”

#### 3.2.3. Interview Duration and Setting

The couple interviews in the present study lasted between 1.5 and 2.5 h. Such extended durations are not unusual for couple interviews, as impulses do not originate solely from the interviewer. Instead, the two partners stimulate and complement each other and jointly reconstruct memories. This dynamic form of conversation required a high level of concentration from all participants. The duration of the conversations was influenced by the participants’ age and the health-related context. Health- and care-related topics often developed only gradually, as they were emotionally sensitive and biographically significant. I frequently observed that the partner in need of care required more time to articulate their thoughts, while the caregiving partner tended to intervene in an elaborating or explanatory manner:

CRP (In the interview excerpts, partners are identified according to their caregiving roles as the caregiving partner (CGP) and the care-receiving partner (CRP). “I” refers to the interviewer.): I’ve fulfilled about 99 percent of my youthful dreams. Traveling. For example, at some point I had to—because my little son (P2: Exactly.) …

[First language]

CGP: That was a bit of his first language, what he said there, that one word. You absolutely wanted, someday, as little Daniel in [large city in Eastern Europe], to go to the Empire State Building. (M: Yes.) And when you say you fulfilled a wish for yourself, that was …

GRP: For example, my father could—Chrysler(P2: Chrysler Building). That was also a dream of mine, to go there and …

CGP: He in turn had a friend who worked next to the Chrysler Building on the 55th floor. He took us there and we were allowed to look at the Chrysler Building as a whole from the office window. What I mean by that is that my husband is very much he’s someone who today also really comes into his own when speaking, because it certainly interests him as well. But otherwise he is actually very reserved in terms of contact, meaning he never takes the first step.

These co-constructive narrative movements, while typical of couple interviews, occupied a particularly prominent place in this specific research field. Health-related limitations also had a direct impact on the conduct of the interviews. In several interviews, it was necessary to reduce the pace, introduce breaks, or respond to signs of fatigue. Although these slowdowns extended the overall interview duration, they proved necessary for establishing a trusting conversational atmosphere. They also enabled the capture of relevant dyadic dynamics and shared processes of meaning-making within the caregiving context. 

Given the extended interview duration, a correspondingly large volume of data was generated. It was therefore necessary to attend to data economy already during the interview process, as not all segments of the conversations could be analyzed in equal depth. A particular challenge consisted in maintaining a clear focus on the research topic, as the interactional dynamics of the couples at times led to broad, time-historical accounts that extended beyond the immediate object of investigation (as can also be seen in the prior interview excerpt). In some interviews, the exchange additionally assumed a more informal conversational structure, resulting in a temporary shift away from the specific care-related thematic framework.

However, these conversational dynamics were shaped not only by the participants themselves but were also strongly influenced by the respective interview setting. The interviews were conducted in the couples’ homes, which co-determined the processes described above. Domestic interview settings are inherently less controllable, as everyday routines and situational disruptions may enter the interview process, for example, when participants temporarily leave the room or attend to household matters. This becomes evident in the following excerpt:

CGP: Oh yes—right! It’s gotten cold, hasn’t it. I don’t know why that is. I’m freezing too.

I: [pours tea] Thank you.

CRP: We’ve got a new heating system at the moment, and we still need to have it adjusted.

I: Oh, I’m not cold.

#### 3.2.4. Observation and Documentation

Observation within the couple interview proved to be challenging. In particular, remaining in interaction while simultaneously observing and documenting what was observed turned out to be difficult. While I was taking notes or listening attentively to an extended response from one partner, micro-interactions between the two partners often escaped my attention. These difficulties were further intensified by the fact that the act of documenting itself intervened in the interactional process. The production of field notes disrupted both staying in contact and the interviewees’ conversational flow, as the sequentiality of the interaction could hardly be captured without substantially interrupting the exchange. Non-verbal communication, such as glances, touches, or gestures of avoidance, was particularly difficult to capture in its entirety, as the partners relied on shared codes that were not always accessible to me as the interviewer. Beyond these challenges of documentation, it became apparent that the analytical attribution of individual observations was also complex, as interactions frequently overlapped or occurred simultaneously. In addition, it was often difficult to clearly assign observations to specific moments of communication. Decisions about what to document and how constituted a powerful act of framing, as I, in my role as interviewer, determined which observations and statements entered the analysis and thus exercised interpretive authority. This selectivity could amplify existing asymmetries within the couple when dominant talk, interruption, or “speaking-for-the-other” aligned with my real-time attentional focus. These processes of selectivity gained additional significance because the caregiving relationship is inherently shaped by dependency, which was reproduced within the interview situation. My observations made visible moments of overwhelm, protective behavior, or advocacy, and could even unintentionally reinforce such dynamics. As an interviewer, I ran the risk of being positioned as an ally or implicit arbiter, for example, when perceiving vulnerability or experiencing feelings of loyalty myself. For instance, I was repeatedly asked to address the husband directly, accompanied by comments such as, “My husband is usually much more open”.

### 3.3. Opportunities in Data Collection

While the preceding section focused on methodological challenges encountered during data collection, the following section outlines the opportunities that emerged from these same conditions. In particular, it highlights how interviewer positioning, interview structure, duration, setting, and observation contributed to the production of rich and differentiated data.

#### 3.3.1. Interviewer Positioning

The decision to conduct the couple interviews alone resulted in a more manageable social setting that facilitated the disclosure of emotionally charged as well as sensitive health- and care-related topics. At the same time, this reduced constellation also heightened the interviewer’s interactional centrality, making it especially important to reflect on how moderating interventions could stabilize or unsettle dyadic power relations. Within the interview situation I designed, a reduced social space emerged in which personal accounts were shared in a more contained interactional framework than would have been the case with two interviewers present. In this dyadic constellation, couples showed a greater inclination to address emotionally charged issues jointly, as attention remained focused on their mutual orientation toward one another. During the couple interviews, it was particularly important to me to maintain an uninterrupted conversational flow, as the partners’ statements were often relationally oriented and developed through joint negotiation processes. Conducting the interviews with a single interviewer facilitated this dynamic by minimizing interruptions and coordination demands. As a result, dyadic interaction remained the central analytical space. Especially in the context of caregiving relationships in later life, which are often shaped by illness-related role shifts and asymmetrical dependencies, this reduced constellation proved advantageous. In addition, the age difference between me, as a younger interviewer, and the older couples encouraged the partners to elaborate more extensively on shared experiences and background knowledge. This supported the reconstruction of their interpretations and facilitated the analytical process. These elaborations sometimes took the form of instructive or corrective positioning (“at your age”), which could both foster narrative richness and reproduce generational hierarchies in the encounter.

#### 3.3.2. Interview Structure and Questioning Practices

While the interactional nature of couple interviews required careful question design (see Section 3.2.2), the thematically structured interview guide proved highly productive for facilitating joint narration. The open-ended questions were deliberately formulated to address both partners simultaneously and to stimulate shared remembering and negotiation. For example, prompts such as “Could you tell me about your relationship history?” or “What shared rituals do you have, and have they changed over time?” encouraged the partners to co-construct their accounts rather than respond individually. The opening question in particular offered early insights into relational dynamics, as it became observable how the partners negotiated who would begin and how the story should unfold (e.g., “You start!”). The thematically structured guide offered both an orienting framework and the necessary flexibility for situational responses. As couple interviews tend to be less linear than individual interviews and narratives often emerge dialogically, the guide primarily functioned as a memory aid during the interview process. Many of the predefined topics were taken up by the couples themselves, while additional aspects not anticipated in the guide also emerged from the dyadic interaction. The sequencing of questions proved particularly important when addressing sensitive or potentially conflict-laden topics. Questions concerning caregiving-related burden or changes in intimacy were deliberately introduced at a later stage of the interview. This structure also facilitated a balance between joint and individual responses. Couple-oriented topics were addressed first, followed by a later focus on individual perspectives. By this stage, role distributions and interactional patterns within the couple had already become clearly observable. In this context, a further characteristic of couple interviews became apparent. My role shifted from that of a primarily questioning interviewer to that of a moderating figure who structured and, when necessary, balanced the interaction between the partners. This role shift shaped both the interview situation and the production of the data and required heightened analytical sensitivity to how moderating interventions influenced the visibility of individual perspectives.

#### 3.3.3. Interview Duration and Setting

Although the extended duration of the couple interviews placed high demands on participants’ concentration, it proved essential for fostering more in-depth conversations. As the interviews progressed, trust gradually developed, so that particularly sensitive topics related to illness and caregiving were often addressed only at a later stage. Moreover, the extended time frame created space for joint processes of remembering and negotiation, in which couples co-constructed experiences. They also introduced aspects not anticipated in the interview guide, such as everyday coping strategies or emotional strains. This extended interaction also created numerous opportunities for interactive observation regarding the couple’s interpersonal atmosphere and their ways of dealing with health-related limitations Over the course of the interview behavior became more everyday-like and a certain “narrative momentum” developed. Even though it became challenging for me at times to maintain a clear focus on the research topic, I experienced these passages as particularly valuable, as they made visible how couples jointly interpreted their health situation and how role changes or burdens were negotiated within the conversation. This became particularly evident in sequences in which questions of dependency and dominance were negotiated in situ:

CP: A partnership is a relationship on equal footing. And then suddenly it shifts, so that one becomes so dependent on the other that a kind of dominance emerges…

CRP: That situation doesn’t exist.

CP: That situation does exist, that the dominance of the one who provides care becomes so strong that you’re no longer a couple.

CRP: And if that were true…

CP: No, that’s fine. But I think you said the first sentence, and for you it was actually already clear…

CRP: We are a couple.

In this exchange, shifting notions of equality, dependency, and dominance are not merely described but interactionally contested. The example illustrates how previously identified patterns, such as correction sequences and “we”-positioning, unfold in situ during extended interviews.

In addition, changes in couple dynamics became observable over the course of the interview, as the extended conversations captured processes rather than mere snapshots. This was particularly relevant in caregiving relationships, where dependencies and power relations are often situational and shifting. Furthermore, interruptions occurred during the long interviews conducted in the home setting, for instance due to the doorbell, telephone calls, brief coordination exchanges, or caregiving activities. Such care-related interruptions became analytically significant, as they made visible how illness and caregiving structured everyday life and temporarily reconfigured attention and participation within the interview situation. The following excerpt illustrates how an incoming phone call briefly interrupts the interview flow and is collaboratively managed by the couple and the interviewer:

[Phone rings]

CGP: That’s Renate, the one who’s taking her husband to the hospital today, so …

I: Ah yes, okay, yes-yes.

CGP: [goes to the phone in the adjacent room]

CRP: There are a few problems …

CGP: [returns] That wasn’t important.

A second example illustrates how these situational shifts were collaboratively managed and reintegrated into the ongoing interaction:

[Doorbell rings]

I: That must be her now.

CGP: Alright then, come on down with me!

I: Yes. Is she coming? So, is she coming now?

CGP: Yes, yes, we’re going now.

I: Ah yes, okay.

CGP: It won’t take long.

The longer the interviews lasted, the more likely such situations were to occur. Conducting the interviews in the home setting further enhanced this analytical gain. In the familiar environment, the couples appeared, from my perspective, less inhibited. This facilitated conversations about health-related limitations, caregiving practices, and changes in intimacy.

In addition, the residential context—including spatial arrangements, material conditions, and patterns of use—became analytically relevant, as it reflected both biographical trajectories and care-related adaptations. Observing how partners positioned themselves within this space provided further insight into relational structures and dynamics. As a result, everyday caregiving practices emerged that would otherwise not have been accessible within the interview itself. These included, for example, assistive devices, pathways through the space, routines, or forms of physical closeness. In particular, spatial arrangements proved to be closely linked to the caregiving situation and to reflect biographical as well as care-related processes of adaptation. For instance, the placement of a care bed in the living room in order to enable the care-dependent partner to participate more fully in everyday life is illustrated in the following interview excerpt:

CGP: It stood here for three years. Instead of the sofa, there was a small care bed placed there, because we said …

CRP: I would have been isolated.

CGP: …I simply didn’t want my husband to lie in the bedroom all day while I was here. He should be here as well. He wasn’t cognitively impaired or anything like that, or, um, so that one could say that in the evenings, if he couldn’t sleep, he could still watch television or continue to participate in family life.

CRP: It wasn’t the case that I was lying there all the time. I slept there (W: yes). then moved into the wheelchair and sat here at the table.

#### 3.3.4. Observation and Documentation

The opportunity to observe within the couple interview opened up a range of methodological possibilities. Attending to non-verbal signals, such as gaze, body posture, physical withdrawal, laughter as a defensive reaction, or shifts in eye contact, supported both the situationally sensitive steering of the conversation and the identification of unspoken topics. When non-verbal cues suggested that something was present “beneath the surface,” I was able to ask follow-up questions carefully and thus access latent or implicit content.

At the same time, observation was central to ensuring the participation of both partners. Particularly in interviews involving care-dependent persons, it helped me assess who required linguistic or thematic support or risked being overlooked. On this basis, I could decide whether to intervene in a moderating way (e.g., “Would you like to add something?”) in order to facilitate a more balanced data collection. Non-verbal signals such as fatigue or signs of overload also enabled me to adapt the interview dynamically, introduce breaks, or postpone topics without risking embarrassment. This form of sensitive, responsive engagement contributed substantially to the development of trust, a sense of safety, and openness among the interview partners.

Beyond the immediate steering of the conversation, the observation of non-verbal communication provided deeper insights into how couples shaped their relationship. This was particularly helpful when narrative or cognitive capacities were limited, as non-verbal cues offered valuable additional information. This became particularly evident in a sequence referring to the routine of applying lip balm upon returning home. It described an embodied caregiving practice that had been observable during the interview and was subsequently interactionally defined as a shared ritual:

CGP: That’s also a ritual.

I: Yes.

CGP: No, lip balm (laughter) When she comes home, she has to…

CRP: Lip balm.

CGP: We’ll start with that, I’ll start with the lip balm first.

I: When you get home.

CRP: Yes, that’s right.

I: Good. Right ritual.

However, even in interviews without such limitations, gazes, mutual forms of address, touch, spatial positioning, and other subtle behaviors allowed for inferences to be drawn about couples’ relationship concepts and internal relationship structures. Such dynamics became especially visible in situations in which roles and power relations came to the fore. For example, when one partner dominated the speaking time and claimed interpretive authority, it was possible to observe how the other partner responded non-verbally or withdrew. In the analysis, this provided valuable indications of how the couple relationship and the caregiving situation were configured.

A further advantage of the couple interview lay in the possibility of immediately identifying corrections, additions, or contradictions. This offered direct insight into shared negotiation processes when partners corrected one another:

I: And where did you meet?

CRP: In front of the university entrance hall, in front of the cafeteria, nineteen…

CGP: Nineteen sixty-two.

CRP: Sixty-three.

Or added to one another’s accounts:

CRP: Well, we used to drive a lot by car, but now I’ve given it up because I…

CGP: …can only see with one eye now.

CRP: Yes, one eye has been gone for quite a long time.

In these moments, I was able to observe both verbal and non-verbal forms of agreement, disagreement, or care. In this way, the potential for distortion due to social desirability was reduced, and a more differentiated picture of the interview situation emerged. Overall, it became evident that in couple interviews, not only what is said but the entire interaction constitutes data, resulting in a particularly rich data corpus.

## 4. Discussion

This study set out to examine the methodological opportunities and challenges of conducting couple interviews with older caregiving couples and to explore how caregiving relations become visible within the interview encounter. The analysis confirms existing research showing that joint interviews provide access to co-constructed illness narratives and dyadic negotiation processes in health contexts [22,23,24,25,26]. However, the present study extends this literature by specifying how such negotiations unfold interactionally. Building on the recurring patterns identified in Section 3.1, the analysis highlights how asymmetries, role shifts, and claims to partnership are enacted and managed within the interview encounter. In this sense, the couple interview provides analytic access not merely to shared narratives but to the micro-processes through which partnership identities are stabilized or defended under conditions of care-related asymmetry.

From a methodological perspective, the study contributes insights into how caregiving relationships in later life become analytically accessible within joint interviews. In these constellations, partnership and asymmetry coexist and must be continuously rebalanced in interaction. While prior studies have emphasized the value of joint interviews for accessing shared illness narratives and dyadic coping processes [22,23,24,25,26], the present analysis focuses on the micro-dynamics of the interview encounter and the interactional production of relational meaning. Rather than treating asymmetries, role shifts, interruptions, or observational constraints merely as methodological challenges, the analysis shows how these interactional constellations may function as epistemic resources for reconstructing relational order.

In contrast to individual interviews, the joint setting made both overt negotiations and subtle micro-dynamics, such as silence, prompting, speaking-for-the-other, or protective interventions, analytically accessible in real time.

These methodological insights are closely tied to the specific context of long-married older couples in Germany living in home-based caregiving arrangements. Interactional patterns such as negotiated role shifts, conflict avoidance, and visible power asymmetries were shaped by long relational histories, age-related vulnerability, gendered generational norms, and structurally embedded care dependencies. Accordingly, the contribution is not a claim of universal interactional regularities, but an analytic specification of what becomes observable under these conditions.

While these conditions limit direct generalization to other couple constellations the findings illuminate broader methodological considerations concerning relational asymmetry, interviewer positioning, and the performative character of joint interviewing. Transferability can be assessed by readers via comparison of interactional conditions (type of dyad, dependency relations, setting, and sensitivity of topics) rather than via demographic similarity alone. The following sections derive practice-oriented implications from this reconceptualization.

### 4.1. Practical Implications for Interviewer Positioning

Based on the present qualitative study involving couple interviews with older caregiving couples, couple interviews conducted by a single interviewer appear particularly well suited when sensitive and intimate topics are addressed and establishing a trusting atmosphere is central. Reduced social complexity facilitates the sharing of personal care- and illness-related experiences while avoiding coordination demands between interviewers. However, the reduced social complexity also concentrates interactional power in the interviewer role. In interviews with older couples, this includes generational positioning, that is, the contrast between biographical junior status and institutional authority. It also includes the interviewer’s capacity to allocate turns, validate accounts, and frame follow-up questions, all of which may shape dyadic power dynamics and the conditions under which emotional disclosure becomes possible. Thematically, this became apparent in negotiations over changing roles and responsibilities, where caregiving partners often assumed interpretive authority, while cared-for partners expressed loss of autonomy.

The analysis highlights the need to develop strategies to address observational blind spots and the high attentional demands placed on a single interviewer. Such challenges are consistent with broader discussions of qualitative interviewing as a complex interactional practice (cf. [33]). This became particularly evident in sequences marked by dominant “speaking-for-the-other,” interpretive authority, or rapid micro-corrections, where non-verbal positioning and subtle withdrawals shaped whose perspective became visible in the joint setting. Marking key passages in the audio recording and producing detailed observation protocols immediately after the interview served as methodological safeguards to address these attentional constraints and reduce the risk of overlooking interactional asymmetries.

At the same time, the analysis indicates that conducting couple interviews with two interviewers can offer clear methodological advantages. In this setting, non-verbal interaction can be documented more comprehensively, as observation and moderation can be shared without interrupting the couple’s exchange. Distributing attention between two interviewers may also help prevent one partner’s dominance from going unchallenged by ensuring that both partners receive validation and opportunities to speak. The presence of a second interviewer also allows for targeted follow-up questions, compensation for memory gaps, and discreet monitoring of recording equipment. However, it may also alter the threshold for emotional disclosure in sensitive caregiving contexts.

Regarding proximity and distance between interviewer and interviewees, the analysis suggests that maintaining a stance of reflexive distance is central in the context of couple interviews on caregiving in later life. While relational proximity facilitated access to sensitive and intimate topics in this study., At the same time, it simultaneously required continuous reflection in order to avoid unintended partisanship and unexamined influence on the production of data. This was particularly relevant in situations of protective interventions or vulnerability, where the interviewer risked being positioned as an ally or implicit arbiter within the dyadic interaction. This positions could potentially shift intra-couple power dynamics by implicitly endorsing one partner’s interpretive authority or claims to vulnerability. This methodological stance resonates with Hitzler’s [34] notion of “deliberate naivety” as a strategy of analytic openness in qualitative research and reflects a tension widely discussed in qualitative interviewing research (cf. [35]).

### 4.2. Practical Implications for Interview Structure and Questioning

Based on the interview dynamics observed in this study, a suitable way to begin couple interviews is to start with an open-ended question that explicitly addresses both partners and leaves room for negotiation regarding who initiates the narrative. Such an approach aligns with methodological discussions of dyadic interviews that emphasize participants’ interaction as a key source of insight [36]. A question such as “Please tell me how you became a couple and how your shared life has developed up to the present day” not only creates a narrative opening but also provides early insight into role distributions, speaker positions, and the internal organization of the couple. Open narrative prompts are known to support self-structured storytelling and reveal interactional positioning within biographical accounts (cf. [37,38]). Such openings therefore function as an early diagnostic window into negotiated speaker roles, “we”-positioning, and patterns of co-narration. Across the interviews, consistently addressing questions to the couple as a unit (e.g., “both of you”) proved effective in signaling openness to contributions from both partners, as also noted in previous methodological work. Nonverbal orientation toward both partners (e.g., gaze and body positioning) further supported balanced participation (cf. [39,40]).

The empirical findings further suggest that addressing individual partners should be postponed until after an initial shared narrative phase, either through follow-up questions or in subsequent individual interviews. In the context of this study, this approach minimized unintended interventions in dyadic narrative processes. This concern has also been discussed in methodological work on dyadic interviewing (e.g., ref. [41]). Intimate or conflict-sensitive topics, such as caregiving burden, conflict, sexuality, or crisis situations, were more productively addressed in later phases of the interview, once sufficient trust had been established. Consistent with Morris [40], separating joint and individual phases reduced interactional strain and enabled couples to manage sensitive disclosures more safely. This staged approach therefore functioned not only analytically but also as a protection against unintended disclosure within the joint interview setting.

For interviewees with cognitive impairments, questions were adapted using plain language [32]. Questions needed to be short, concrete, and narratively accessible. In the present study, these adaptations were crucial for enabling equal participation by both partners.

A preliminary qualitative pilot study (e.g., group discussions or expert interviews) proved valuable in identifying relevant thematic areas and formulating narratively compatible questions. In this research field, this step improved the fit and validity of the interview guide (cf. [31]). Where resources allow, rehearsing moderation in advance may facilitate confidence in handling recurring dyadic interaction patterns.

### 4.3. Practical Implications for Interview Duration and Setting

Consistent with prior research on dyadic interviewing [41] and Morgan’s [13] emphasis on interactional complexity, the findings confirm that couple interviews require extended time due to co-narrative remembering, repair sequences, and negotiated interpretations. Adequate scheduling, including time buffers and the option for breaks, proved essential, particularly with older caregiving couples.

In conducting dyadic interviews with older couples, reduced narrative pace and situational interruptions required flexible moderation. Interactional density often increased over time, as corrections, completions, subtle contradictions, and shifting speaker positions became more visible once trust was established. Given the large volume of material generated, structuring interview guides around clearly defined core and optional topics helped maintain analytic focus without suppressing emergent interactional dynamics [39,40]. Particularly relevant questions were placed early in the interview, while gentle redirection proved effective when extended biographical digressions risked displacing the research focus.

Conducting interviews in couples’ home environments offered distinct methodological advantages. The home functioned as a natural interactional setting and as a “care landscape” in which spatial arrangements, assistive devices, and everyday routines rendered relational proximity, dependency, and embodied coordination observable (cf. [42]). Partners’ spatial positioning frequently indicated patterns of support, withdrawal, or dominance. For this reason, interventions in spatial arrangements should generally be avoided, as domestic space forms part of established interactional orders (cf. [42]). Only in cases of pronounced asymmetries may careful adjustments be considered.

### 4.4. Practical Implications for Observation and Documentation

Observation in couple interviews is necessarily selective, as moderation, steering, and interactional participation must be managed simultaneously. This aligns with the concept of the “observing interview” [43], which emphasizes methodological focus rather than completeness. In line with Valentine’s [39] argument that observation in couple and household interviews requires analytic prioritization, this study treated observation as a distinct methodological layer oriented toward recurring interaction markers rather than exhaustive documentation.

Analytically relevant markers included non-verbal interaction patterns (e.g., gaze shifts, touch, withdrawal), negotiated speaker roles, “speaking-for-the-other,” repair and correction sequences, protective interventions, and spatial positioning within the domestic “care landscape” (cf. [42]). Focusing on such recurring patterns proved more productive than attempting comprehensive documentation and supported both interview conduct and subsequent analysis [30].

Methodological work on multi-person interviews highlights that different configurations of joint and separate interviewing entail distinct epistemic consequences [44]. Where co-moderation is not feasible, systematic post-interview documentation is essential. Observational notes were supplemented immediately after the interview with memory protocols and reflexive accounts of interviewer positioning, capturing interactional dynamics, selective decisions, and moderation interventions. Recording time markers, such as moments when one partner temporarily left the room, enhanced analytic precision. While video recording may facilitate fine-grained reconstruction of embodied micro-interaction [45], its potential intervention effects require careful ethical consideration.

Finally, interviewers themselves form part of the interactional order. Situations in which interviewers were positioned as moderators, allies, or implicit arbiters became analytically informative rather than merely disruptive. Continuous reflexive documentation of such positioning constitutes a central component of transparent interpretation in dyadic research [31]. In sum, the methodological findings can be systematized into a three-part practice-oriented toolkit. It integrates relationally staged interviewing strategies, moderation practices for addressing interactional asymmetries, and analytic observation and documentation markers. Together, these elements render the couple interview a more systematically deployable method in dyadic qualitative health research.

## 5. Conclusions

The opportunities and challenges of couple interviews with older caregiving couples, as reconstructed in this study, suggest that this method constitutes a distinct and analytically productive instrument in qualitative health research.

The opportunities and challenges of couple interviews with older caregiving couples, as reconstructed in this study, demonstrate that this method constitutes a distinct and analytically productive instrument in qualitative health research. The analysis showed how couple interviews enable researchers to access the joint construction of reality, negotiated role shifts, and the everyday enactment of caregiving within long-term relationships. In the context of later-life care, the interview itself became a space in which tensions between partnership and dependency were interactionally negotiated and relational equality was defended or recalibrated under conditions of vulnerability.

At the same time, conducting and analyzing such interviews, particularly with older, health-impaired couples, places high demands on planning, moderation, observation, and reflexive positioning. The study therefore underscores the need for more systematic methodological engagement with couple interviews, especially regarding the analysis of dyadic interaction, mutual corrections, non-verbal communication, and the reconstruction of relational power and dependency structures. Capturing the “in-between” as an analytical level requires explicit conceptual and methodological attention.

For healthcare practice, insights into couples’ lifeworlds generated through joint interviews are particularly relevant. In home-based care arrangements, caregiving is embedded in long-standing relational histories and negotiated identities. Couple interviews reveal how care services intersect with these relational dynamics and may therefore support more relationship-sensitive forms of counseling and support. By demonstrating both the epistemic potential and the methodological demands of this approach, the study contributes to a more differentiated understanding of caregiving relationships in later life. It also argues for a more confident and reflexive use of couple interviews in qualitative health research.

## Data Availability

The data presented in this study are derived from a previously conducted qualitative study with older couples in caregiving relationships. Due to ethical considerations and participant confidentiality, the interview data are available on request from the corresponding author.
